# 

**Published:** 1892-12

**Authors:** 


CONTENTS.

Vol. 39. No. 12.

| | PAGE. |
|---|---|
| Thanksgiving | 265 |
| Cases of Poisoning | 266 |
| An American in India | 270 |
| Ascending Mount Orizaba | 274 |
| Food Constituents | 276 |
| Modern Wasp Waists | 277 |
| Be Straight | 278 |
| The Couch in a Cosey Room | 279 |
| Diphtheria and Croup | 279 |
| Some Eminent Misers | 280 |
| Florence Nightingale | 282 |
| A Curious Custom in Chili | 282 |
| Gout | 283 |
| Vice Sets Its Seal | 284 |
| Miscellaneous | 284 |
| Literary | 287 |
| A Captive | 288 |

INDEX TO ADVERTISEMENTS OF HALF A PAGE AND OVER.

| | PAGE. |
|---|---|
| Dr. Jaeger's Sanitary Woolens | I |
| Christian Life | I |
| Newspaper Advertising | II |
| The Rip Van Winkle Rocker | IV |
| Ayer's Sarsaparilla | V |
| Hall's Journal Premiums | VII |
| Washington Improvement Co. | VIII |
| Magic Fuel | IX |
| Herba Vita | X |
| Royal Baking Powder | XI |
| Buffalo Lithia Water | XI |

